# The genetics and molecular biology of neural tumors

**DOI:** 10.1038/sj.bjc.6605524

**Published:** 2010-02-16

**Authors:** S Brandner

**Affiliations:** 1 Division of Neuropathology and Department of Neurodegenerative Disease, UCL Institute of Neurology, Queen Square, London WC1N 3BG, UK

The rapid development of molecular biology techniques in the last three decades has seen a powerful shift from classical cytogenetics to chip technologies and deep sequencing of whole tumour genomes. Today, several relatively low-cost technologies enable us to study whole tumour genomes and gather information about a tumour's genomic, transcripteomic and proteomic profile almost overnight. More technologies, such as chromatin, methylation and micro-RNA chips, will soon become mainstream analysis in many pathology laboratories.

Against this backdrop, this book summarises cytogenetic information on neural tumours, and hence concludes an era of molecular profiling of cancer.

The book title promises to cover neural tumours. Slightly unusually, however, the authors excluded several important entities of bona fide neural tumours, such as gliomas, neuronal tumours and mixed neuronal-glial tumours, which form a large proportion of brain tumours. Instead, ‘non-neural’ tumours such as meningiomas, hemangioblastomas and paragangliomas are included in this book.

The book therefore essentially covers nerve sheath tumours, meningiomas, hemangioblastomas, neuroendocrine tumours as well as a mixed group of neuroblastic and primitive neuroectodermal tumours. The book is written very systematically, in that every chapter contains an overview on each tumour type and then describes the most relevant chromosomal changes. Every chapter also contains very extensive and very well referenced tables. The meticulous listings of all chromosomal abnormalities that have to date been reported for these tumours are probably unmatched in the literature. In addition, the key literature is summarised by a brief one-line summary that is compiled to a large table with references. Further, this cytogenetics reference book shows karyotype reprints, comparative genomic hybridisation schematics and the typical histological appearance of those tumours. There is also a very systematic description of molecular signalling pathways and Kaplan–Meier survival curves to illustrate the relevance of a certain mutation to the prognosis of the tumours.

When I started reading this book, I was full of expectations about the molecular genetic changes in tumours and anticipated furthering my understanding of their pathogenesis. Having seen in the introduction a reference to a possible comparison to animal models, I expected a correlation of (mostly, but not exclusively, mouse) model systems with the human counterpart. The book meets my expectations regarding the description of signalling pathways relevant to human tumours, but I was disappointed to find very little reference to animal models, which have significantly contributed to the advancement of our understanding of tumour pathogenesis. Another disappointing feature of this book is the lack of colour prints. Although this can be tolerated for histological photographs, which are not a focus of this book, a three-colour illustration of pathways and other schematics would have improved the overall appearance considerably. Moreover, some of the halftone reproduction is of poor quality and I am somewhat surprised about the lack of attention to the drawings. For example, the pathways are often not presented very clearly, making it sometimes difficult to understand and to follow the positive or negative feedback mechanisms. Also, the relevance of gain of function or loss of function should have been illustrated in this context.

Although the tables are useful as a reference, they are sometimes very hard to read, in particular with multiple chromosomal gains and losses. A table in which each box is reserved for a specific chromosome (semi-graphical representation) might have improved the legibility.

Beyond these issues that relate to graphical editing and may be regarded as less important by some readers, the lack of reference to most recent technologies such as SNP analysis, deep sequencing and expression array analysis with relation to the existing cytogenetic findings appears perhaps more significant. Meanwhile, there are a number of databases that are publicly accessible and from which a wealth of data can be downloaded and analysed. An overview would have been helpful in delineating the future potential of these novel technologies.

In conclusion, this volume surprisingly omits most primary CNS tumours (except medulloblastoma), which is slightly confusing as these are typically and primarily referred to as ‘neural tumours’. The book serves well as a data repository with an enumeration and listing of karyotypes, chromosomal aberrations and a detailed description of the pathways involved in the pathogenesis of these tumours. What I miss in this book is a reference to model systems, many of which have been key to the understanding of the histogenesis and molecular pathogenesis of many human brain tumours. This deficiency becomes particularly apparent in the chapter ‘medulloblastoma’ for which a wealth of mouse models has been generated over the last decade. A thorough and systematic comparison of the pathways and cells involved in the pathogenesis would have been very informative. Also surprisingly, the authors have referred to the old WHO classification (2000) rather than referring to the revised classification of 2007, which has seen important changes to the terminology and classification of primitive neuroectodermal tumours, neuroblastoma and medulloblastoma.

I recommend this book as a reference manual for cytogeneticists, but may not recommend it to my students or research fellows who not only need to understand the correlation between molecular events and tumour pathogenesis but importantly also need inspiration and vision for their own research.

